# Prevalence of hidden carbon monoxide poisoning in auto service workers; a prospective cohort study

**DOI:** 10.1186/s12995-018-0214-9

**Published:** 2018-11-06

**Authors:** Oğuzhan Bol, Serhat Koyuncu, Nurullah Günay

**Affiliations:** 1 Health Science University Kayseri Training and Research Hospital Emergency Medicine Department, Kayseri, Turkey; 2 Gaziosmanpasa University Emergency Medicine, Tokat, Turkey; 3 Erciyes University Emergency Medicine, Kayseri, Turkey

## Abstract

**Background:**

Carbon monoxide (CO) is formed as a result of the incomplete burning of hydrocarbon-containing fuels such as natural gas, coal, liquid petroleum gas, and wood. CO is a colorless, odorless, and poisonous gas that produces various acute and chronic effects in CO-exposed people. In this study, we aimed to measure CO levels in auto care repairmen with chronic CO-related illnesses using a serial, non-invasive method.

A prospective cohort study.

**Methods:**

A total of 99 people from six different auto-repair services were included in the study. Carboxyhemoglobin (COHb) levels were measured at four different times with 2-hour intervals starting at 08:00 AM. Data concerning employees’ ages, working hours, smoking statuses, and types of home heating fuel were collected. A control group of 100 cases was created based on this data. The measurements were done on the control group in the morning with a Masimo Rad-57 CO-oximeter.

**Results:**

The highest mean (± SD) COHb value was 7.04% ± 3.32% after the third measurement. The mean value for the control group was 1.61% ± 1.43%. A statistically significant difference between the groups was found for each value.

**Discussion:**

We determined that the risk of being affected by CO is high in buildings in which the auto services were located. The effects of chronic or prolonged exposure to low amounts of CO were found to be ambiguous. However, in some studies, it was found that low-grade CO exposure could lead to coronary artery disease and some neurological complications. Therefore, it is necessary to be careful about the health of employees who have been exposed to CO.

**Conclusions:**

We concluded that there is a need for more detailed studies concerning chronic CO poisoning. Also, in workplaces in which there is high exposure to CO, proper workplace safety measures should be taken to reduce this gas’s harmful effects to employees.

## Objectives

Carbon monoxide (CO) is a colorless and odorless toxic gas [1, 2] that is produced mainly as a result of incomplete combustion of hydrocarbon fuels such as natural gas, coal, liquefied petroleum gas, and wood [1, 3, 4]. In people’s daily lives, environmental CO exposure is typically < 0.001% or 10 ppm (ppm) [1, 2, 5]. While an adult cigarette smoker is exposed to an estimated 400 to 500 ppm of CO during active smoking, automobile exhaust may contain around 100,000 ppm CO as measured inside a closed garage [1, 4, 6]. Based on these values, the Air Quality Guidelines for Europe (Second Edition), which were published by the World Health Organisation, stated that a COHb level of 2.5% should not be exceeded depending on the CO levels in the environment and duration of exposure. Ambient CO values that are required to reach this maximal acceptable value of serum COHb are presented in Table 1 in units of mg/m^3^ and ppm [5].

**Table 1:** Air Quality Guidelines for Europe, Second Edition published by the World Health Organisation (WHO) state that a carboxyhemoglobin (COHb) level of 2.5% should not be exceeded as determined in ambient carbon monoxide (CO) concentrations after a given exposure period

CO concentration (mg/m^3^)	CO concentration(ppm)	Exposure Time (Minute)
100	90	15
60	50	30
30	25	60
10	10	480

Reports on the acute phase of CO gas exposure are prevalent, whereas chronic exposure has rarely been reported. Chronic CO exposure is considered to be more prevalent and much more closely associated with increased morbidity and mortality than previously accepted or assumed [1, 2]. Chronic exposure is defined as prolonged exposure to low amounts of CO [7]. Chronic exposure can be an underlying cause of nonspecific complaints such as headaches, nausea, vomiting, and asthenia and dizziness in addition to clinical manifestations, including cerebellar dysfunction and acute coronary syndrome [1, 2].

CO poisoning is quite common among the population but there are certain occupational groups who carry a greater risk of exposure to this gas due to their occupation [8]. These occupational groups include auto service maintenance personnel, exhaust pipe repairmen, chefs using ovens or grills in which charcoal is used, gasoline pumpers, and traffic policemen. Although chronic exposure of these people to CO gas has been reported previously, insufficient scientific data exist concerning possible chronic daytime CO exposure of these individuals, particularly the ones who work in auto service centers [9]. Therefore, autoworkers were selected as the target occupational group for this study.

## Study design

A prospective cohort study was conducted.

## Methods

This study was conducted between 10.12.2012 and 10.01.2013 after obtaining approval from the local and ethics committees (Erciyes University Ethics Committee approval date: 04.12.2012 and the Decision Number: 2012/701). A total of 99 male employees working at six different car service centers in Kayseri were included. For each participant, a total of four serial daytime CO measurements were made, and COHb levels were recorded on the same day starting at 08:00 AM (beginning of office hours) followed by measurements at 10.00 AM, 12.00 PM and 14.00 PM. The measurements were done at the workplace and were performed in a noninvasive manner with Masimo Rad-57 Oximeter (Masimo Corporation, Irvine), which has an accuracy of 3% according to the manufacturer. The device has the same format as the commonly known finger pulse oximeter. Additionally, workers were asked questions such as whether they smoked, what kind of fuel they used to heat their home, how long they had been working, and whether they had recently complained of a headache, weakness, nausea, insomnia, and/or confusion (delay in understanding what has been said). In order to be eligible for this study, the car service had to be a closed area and heated without using an energy source that potentially emits CO (such as charcoal/ wood or similar fuels). Inclusion criteria for autoworkers included a minimum of 1 month of work experience at their current auto service, > 18 years of age, and working in the auto service’s mechanic division.

A control group of 100 subjects who were matched with the study group with regard to age, gender, and the use of a stove or natural gas for heating was included. The control group consisted of the relatives of patients who were admitted to the emergency department triage unit between 08:00 AM and 10:00 AM. As with the study group, the ones with a chronic illness were excluded by evaluating them with routine histories and physical examinations. Also, initial COHb values of the study group were compared with those of control group.

The following protocol was applied for autoworkers with regard to the measurement of COHb levels: The threshold COHb value was determined as 10% in smokers and 5% in non-smokers, and any value exceeding this was considered an indication of CO poisoning. Threshold levels of COHb poisoning were determined with reference to previous studies [10–12]. Workers with symptoms of acute poisoning were admitted to our emergency department and managed by emergency medicine physicians. An additional blood sample was obtained from these individuals, and patients were treated if toxicity was detected.

Data from patient and control groups were recorded into the licensed SPSS (IBM, ver21, United State) software package. Categorical variables were compared using the chi-square exact test. Normal distribution of numerical variables was checked using the Shapiro-Wilk test. Comparisons between measurements were performed by one-way repeated variance analysis (ANOVA) and multiple comparisons were performed using the Bonferroni test. Two independent sample t- and one-way ANOVA tests were used for intergroup comparisons. The relationship between numerical variables was evaluated by Pearson’s correlation analysis. A *p*-value < 0.05 was considered statistically significant.

## Results

**Fig. 1:**
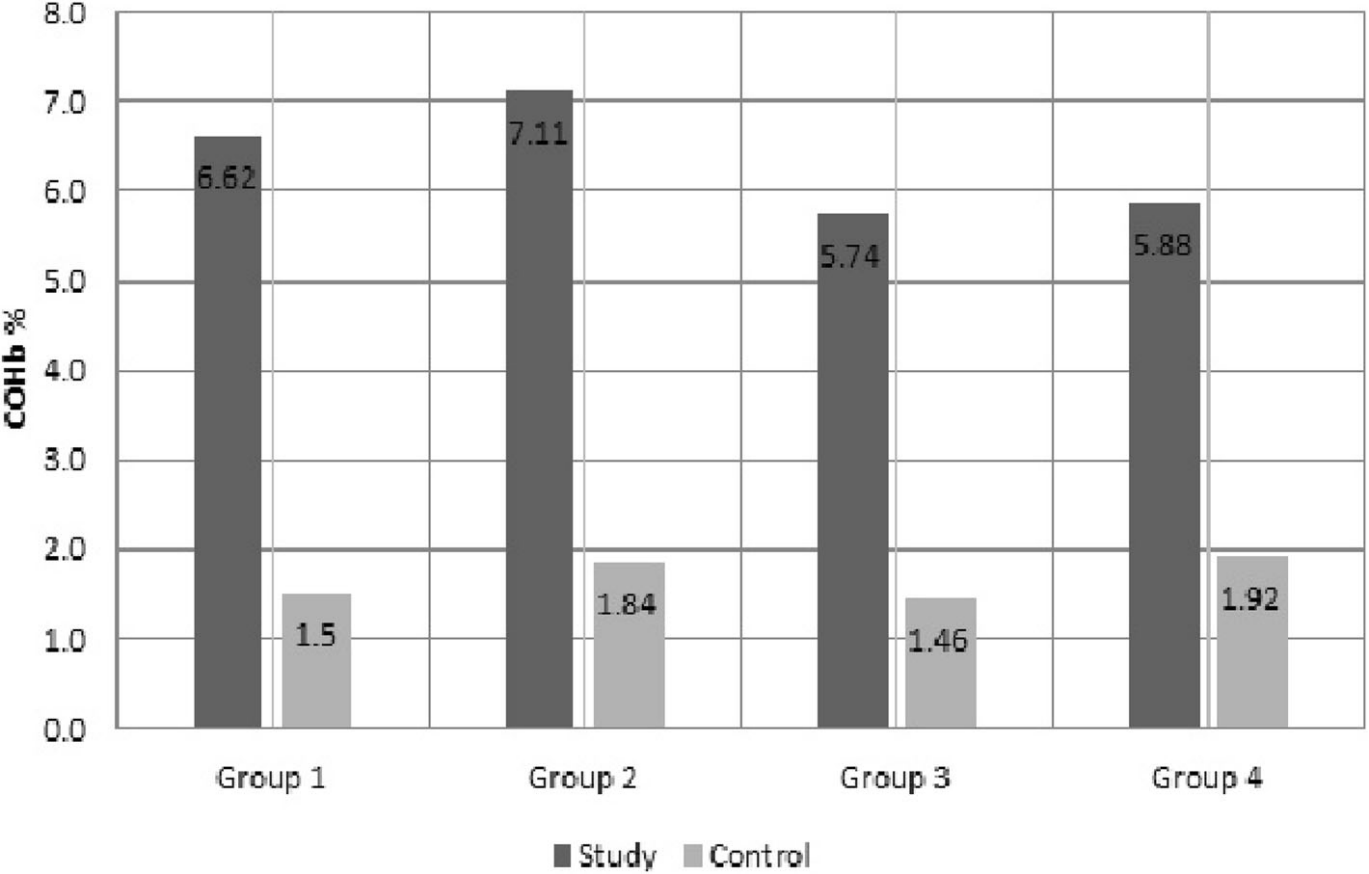
Carboxyhemoglobin values in workers and controls, stratified by smoking status and home heating system. **Group 1:** Smokers who use natural gas for heating (study; n:35, control; n:36, min-max value worker and control group; 0–18 and 0–4, 95% CI of study group 5.68% to 7.55%). **Group 2**: Smokers who use charcoal for heating (study; n:19, control; n:19) (study; n:19, control; n:19, min-max value worker and control group; 1–15 and 0–4, 95% CI of study group 5.84% to 8.38%). **Group 3:** Non-smokers who use natural gas for heating (study; n:32, control; n:32, min-max worker value and control group; 0–13 and 0–4, 95% CI of study group 4.76% to 6.72%). **Group 4**: Non-smokers who use charcoal for heating (study; n:13, control; n:13, min-max value worker and control group; 0–14 and 0–4, 95% CI of study group 4.34% to 7.42%)

Of 14 auto service centers with a certificate of authorization for operating in the city of Kayseri, six were included in the study. In this study group of 99 workers, duration of work experience in the field of occupation ranged between 3 months and 25 years with a mean ± SD of 15.16 ± 9.9 years. Their ages ranged from 18 to 45 years (mean ± SD, 31.59 ± 8.98). For the control group, ages ranged between 19 and 45 years (mean ± SD, 30.4 ± 7.4). COHb values of the groups, smoking statuses, and types of heating are shown in Fig. 1. In the study group, the highest and lowest COHb values were 18 and 0%, respectively. In the control group, the corresponding COHb values were 4 and 0%, respectively.

In our study, 10 smokers (18%) with a COHb value of > 10% and 33 non-smokers (73%) with a COHb value > 5% were detected at any time of measurement.

When we analyzed the results individually, the highest COHb values of 15, 15, 18, 13, 11 and 15% were obtained from six different auto service centers after four measurements. The total number of workers who had at least one measurement exceeding the pre-specified threshold exposure level of 5 or 10% based on smoking status were nine, five, 10, five, six, and eight, and a total of 43 workers were found to have threshold levels that exceeded this level.

Among workers, the highest mean (± SD) COHb value of 7.040 ± 3.32% was obtained during the third measurement. The mean (± SD) value was 1.61 ± 1.43% in the control group. Other values are shown in Table 2.

**Table 2:** Mean carboxyhemoglobin [%] in the control and study groups at different time points

	08:00	10:00	12:00	14:00
Study Group	5.21 ± 3.23	6.44 ± 3.34	7.04 ± 3.32	6.64 ± 3.28
Control Group	1.61 ± 1.43	–	–	–

Differences between four measurements and the relationship of the values obtained with smoking and the type of heating were also analyzed for the study group. Values obtained from the control group were statistically analyzed together with the initial values of the study group.

Within the study group, there was a significant difference in COHb levels from all four measurements.

This difference between measurements was also significant when each sub-group in the control and test subject groups was compared: smokers (*p* < 0.001), nonsmokers (*p* = 0.004), charcoal users (*P* = 0.004), and natural gas users (p < 0.001) employees.

**Fig. 2:**
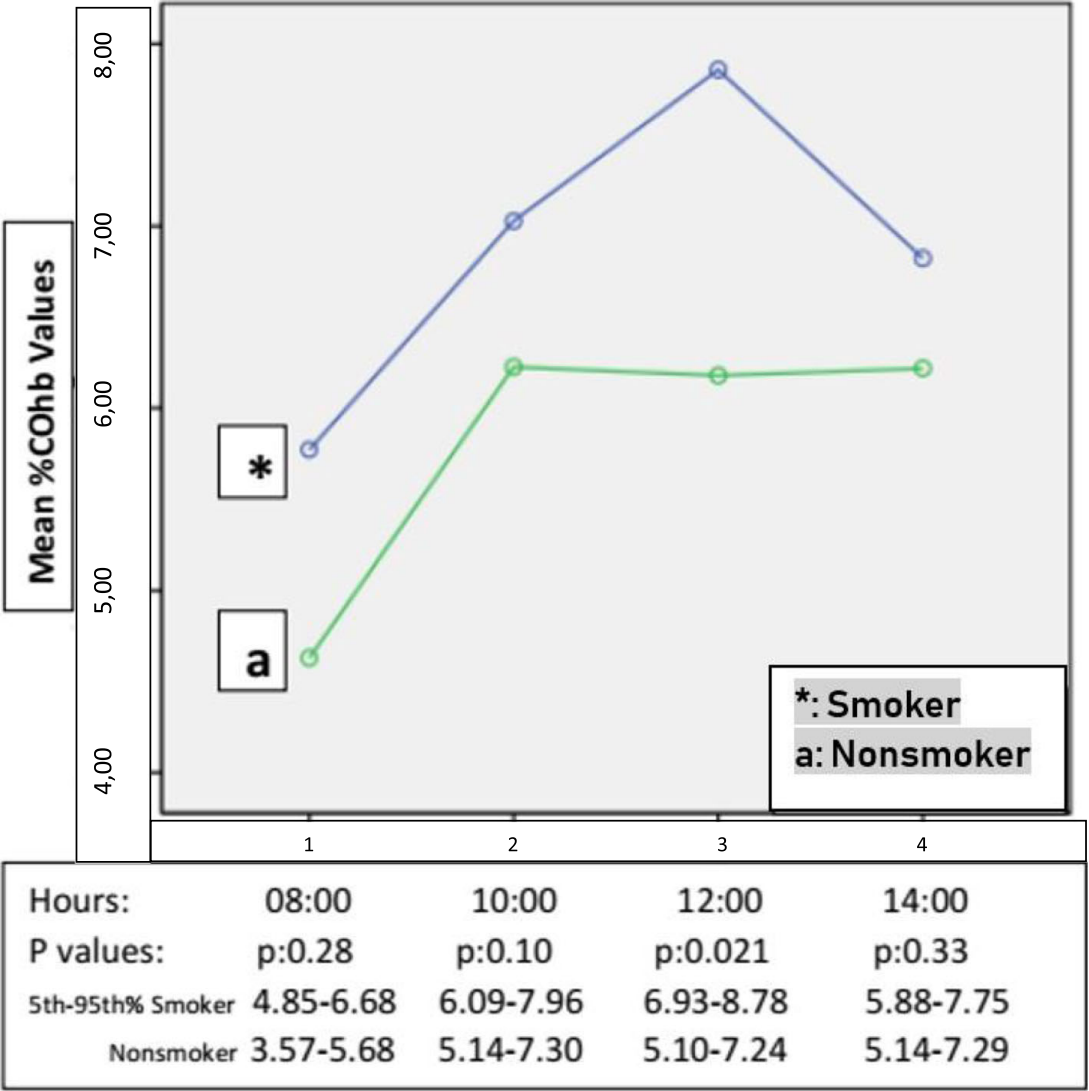
Statistical difference in mean carboxyhemoglobin between the 4 different measurements times in the study group

For the study group, a statistically significant difference in mean COHb was obtained at the third measurement at 12:00 PM was detected between smoking and non-smoking employees (*p* = 0.021). Other measurements did not show a statistically significant difference; *p* = 0.28 at 08:00 AM, *p* = 0.10 at 10:00 AM, and *p* = 0.33 at 02:00 PM (Fig. 2).

For the study group, a statistically significant difference was not found when workers using natural gas for heating were compared with workers using charcoal.

A statistically significant difference was observed between all measurements of study group and control group (*p* < 0.001).

When we analyzed smokers in the study and control groups, a statistical difference was not found between COHb values. Nonsmokers in study and control groups did not show significant differences regarding the measured COHb values either. (Table 3).

**Table 3:** CoHb values in smokers and non-smokers in the study and control groups

	95% Confidence Interval for Mean Lower Bound/Upper Bound	Mean, Min-Max
CoHb values in smokers (n:109)	2,9566/ 4,2361	3,5963, 0–17
CoHb values in non-smokers (n:90)	2,6048/ 3,7285	3,1667, 0–10

There was no statistically significant difference in measured COHb values between study and control groups regarding those who used natural gas for heating. Similarly, a significant difference was not observed in measured COHb values between study and control groups for those who used charcoal for heating.

## Discussion

We believe that CO poisoning cases other than acute poisoning are even more remarkable because of the difficulty of detecting such cases and the impossibility of finding them without definitive proof. Diagnosis of chronic CO poisoning in patients admitted to emergency services is still not easy as the presence of similar clinical presentations cannot be excluded. CO gas may harm any cell in the body which is dependent on oxygen regardless of its amount, causing a range of conditions from acute coronary syndrome to detectable damages in the brain [1]. Based on this knowledge, we planned to conduct a study with autoworkers due to their involvement in an occupational group that is known to be associated with risk factors for CO-related chronic poisoning and because of the inability to diagnose such cases in the emergency room.

Even the highest and lowest COHb values (18 and 0%, respectively) that are found in members of an occupational group who had a risk factor for chronic CO exposure alone supported our hypothesis that chronic and even acute effects of CO poisoning can be seen in autoworkers. That is to say, breathing CO of 18% itself calls for suspicion of an acute poisoning that requires urgent treatment. However, we believe that the aforementioned complaints are often overlooked and not verbally expressed in daily life and/or thought of as signs of other ailments. Another possibility is that even if employees are chronically exposed to CO, they might spontaneously recover from its effects before they decide to seek medical attention, which would preclude a proper diagnosis at the time of presentation. Our research may be valuable for demonstrating the possible effects of CO exposure in the sense that measurements should be performed right after exposure and at the site of exposure. On the other hand, detection of a COHb value of 0% was interpreted as a lack of absolute homogeneity in a given auto service and accordingly, autoworkers may be exposed to CO at different levels depending on their location and the work they perform in the auto service.

By examining the number of workers with values exceeding the threshold, one might assume that these workers are chronically affected by CO gas and beyond that, they are actually on the brink of acute poisoning. Since elevated COHb values were detected in 43 workers in the study group, all of them were invited to be examined in our emergency department because of the possibility of acute poisoning. Among these workers, 33 accepted the invitation and came to the emergency department after work followed by diagnostic testing and treatment. During this time, invasive COHb measurements were conducted to confirm exposure to CO gas, and 26 workers who were found to have serum COHb values exceeding the pre-determined threshold were administered normobaric oxygen treatment at a flow rate of 2 to 4 L/min for nearly 1 h. The absence of complaints at the time of ER admission in the other workers with normal serum COHb values, which were detected by invasive measurements, suggested that workers might have experienced clinical relief as a result of being away from the CO source and cleared the CO gas during the time period after leaving the workplace until admission to the emergency department. Workers were discharged after evaluation of both laboratory parameters and symptoms (if there were any) as observed by pre- and post-treatment monitoring, and they were considered to be affected by CO gas. Despite the presence of a control group, baseline COHb value of the workers was also determined using their initial measurements. Even when the control group was not considered, readings in the study group obtained at 10:00 AM, 12:00 PM, and 02:00 PM were found to be significantly elevated compared to their baseline values. Thus, during the day, workers continued to be incrementally affected by CO gas at 2-h interval beginning from the time (08:00 AM) they started working. As a study objective, smoking habits and types of home heating were also examined in detail in order to demonstrate the presence of an occupational risk factor for chronic CO exposure after taking these parameters into account.

Employees usually do their job in a closed area and remain continuously close to a CO source, especially in the presence of running motor vehicles. In our study, a possible CO source was considered to be the auto service center itself. Although there were smokers among the employees, we observed that they avoided smoking in closed areas of all areas of the car services. Thus, the car service was considered to be the CO source among nonsmokers.

While subsequent COHb readings were elevated compared to baseline, a statistically significant increase that was found at the third measurement of smokers was not due to smoking alone. This elevated third reading was considered to be associated with increased admission of more motor vehicles during that time period to the car service. Another interesting point was that the third reading roughly coincided with the time of the employees’ lunch break. During this break, smokers were observed to smoke cigarettes more frequently.

A comprehensive study similar to ours was conducted by Kelman et al. who measured the serum COHb values of 61 employees working at 35 Leicestershire garages. A single sample was obtained in that study, which found elevated COHb values in 20% of smokers and 43.8% of non-smokers. Their threshold values were the same as ours (> 10% for smokers and 5% for nonsmokers); however, the authors did not take the difference between invasive and non-invasive measurements into account (11). As an interesting finding in our study, elevated COHb values were obtained among non-smokers in contrast to that study. Smokers are exposed to a certain amount of CO on a daily basis as a result of to their habit, but nonsmokers are only exposed to CO at their workplaces. Persistently high CO exposure among nonsmokers and continuous near-threshold CO exposure among smokers puts these individuals at a higher risk for chronic CO poisoning. Also, our study used serial measurements instead of a single reading. Since CO exposure was monitored among both smokers and nonsmokers throughout the day, we believe that our study demonstrates the danger of chronic exposure more accurately.

In a study by C. M. Castleden et al., COHb values obtained from smoking and non-smoking employees working in different occupations were analyzed. Smokers were found to have a significantly higher mean COHb value (5.8%) in comparison to that of nonsmokers (1.3%) [13]. In the present study, smoking and non-smoking employees differed only with regard to the values of the third measurement.

We conducted a literature scan in order to identify the studies that focused on a link between CO exposure and the type of home heating used by employees and could not find one. When we analyzed CO values according to the type of household heating, a statistically significant association was not found. Before initiating the study, we expected to see different results among employees who used charcoal for home heating. We also considered that a possible CO elevation cannot be identified among employees based on these data during the time period between leaving the house and starting of jobs.

We obtained a statistically significant difference between the data that were obtained from the study and control groups. This major difference that we observed between control group consisting of individuals who left their living environment and were admitted to hospital in the early hours and the study group might actually indicate the risks that the study group encounters on a daily basis. Workers in the study group are exposed to a considerable amount of CO not only for a short-term but also throughout their office hours and even for the majority of their lifetime. Moreover, we believe that these individuals might experience a diminished quality of life over long-term, especially after considering their smoking habits.

In one study, G. Kocasoyet al. compared CO and COHb before and after working values obtained from 72 employees and those of a control group (smoking and non-smoking), who worked at the Istanbul Bosphorus Bridge tolls. In comparison to the control group, CO and COHb values showed a statistically significant difference between smoking and non-smoking employees. A significant difference was found between smoking and non-smoking employees with regard to CO and COHb values before and after working hours [14]. In the present study, the opposite results were obtained. There was a statistically significant difference between our study and control groups with regard to CO values; the study group had a significantly elevated CO exposure. However, we did not find a statistically significant difference between the study and control groups when we examined smoking and non-smoking employees separately.

In a study by M. Abdollahi et al., bus drivers working in the city center were found to have significantly higher blood COHb values (4.19% ± 0.65%) compared to those of control group (1.09% ± 0.27%) [15]. These findings are similar to ours.

S. Nizaet al. conducted a study with employees working in toll bridges and offices in the surrounding area and found higher COHb levels among toll bridge workers than office employees. They observed an increase in the COHb levels, especially during the rush hour when the traffic intensity increased [16]. These findings are consistent with ours.

What these three studies have in common is the presence of elevated COHb values among employees who are exposed to intense exhaust gas at their workplaces. These results are also consistent with ours. In this study, COHb levels of the study group were significantly higher than the control group. These data show that individuals working in garages are persistently exposed to the effects of CO when compared to people living under normal environmental conditions.

To this date, studies have investigated the effects of chronic CO exposure in different occupational groups. However, no studies using serial measurements have been conducted that have explored possible chronic CO exposure among auto service workers. As a result of the data presented in this study, it can be concluded that auto workers were chronically exposed to a substantial amount of CO gas during their working hours irrespective of their smoking habits and from the carbon-based fuels they use for heating their homes. At this point, as researchers, we are left in the dark regarding long-term clinical manifestations that may occur in the participating auto workers. A study of the long-term clinical manifestations might be an interesting starting point for enthusiastic researchers who wish to conduct further extensive studies about this matter.

## Limitations

The most important limitation of this study was the lack of air sampling from the environment around the study or the control group.

## Abbreviations

CO: Carbon monoxide
COHb: Carboxyhemoglobin
Ppm: Per million
